# Supplementary material for: New Strategy for the Design of Anti-Corrosion Coatings in Bipolar Plates Based on Hybrid Organic–Inorganic Layers
Source: Molecules. 2023 Apr 6;28(7):3279. doi: 10.3390/molecules28073279 (PMC10097085; doi:10.3390/molecules28073279)
Supplement: Supplementary file 1 [file molecules-28-03279-s001.zip › molecules-2312079-supplementary.pdf]

Supporting information for

New strategy for the design of anti-corrosion coatings in bipolar plates based on hybrid organic-inorganic layers

Xiaoxuan Li [a]， Wenjie Sun [a], Yuhui Zheng [a], Chenggang Long [b]*, Qianming Wang [a,b]*

[a]. School of Chemistry, Guangzhou Key Laboratory of Analytical Chemistry for Biomedicine, South China Normal University, Guangzhou 510006, PR China.

[b]. Ruide Technologies (Foshan) Inc. Foshan, Guangdong, 528311, China

*** Corresponding Author**

Tel.: 86-20-39310258, Fax: 86-20-39310187. E-mail: long@ruideinc.com; qmwang@scnu.edu.cn.

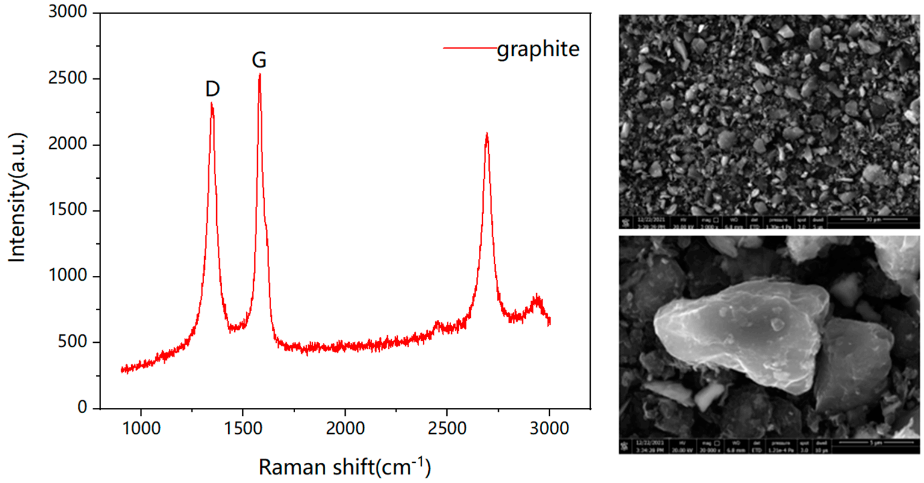

Fig. S1 Characterization of graphite powder: Raman Spectrum and SEM image of graphite powder

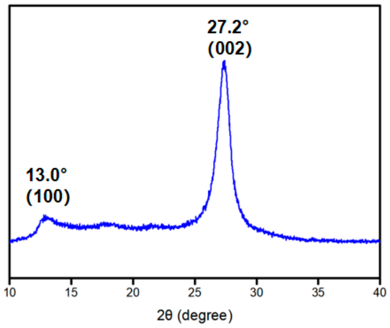

Fig. S2 X-ray diffraction (XRD) patterns of carbon nitride nanosheets

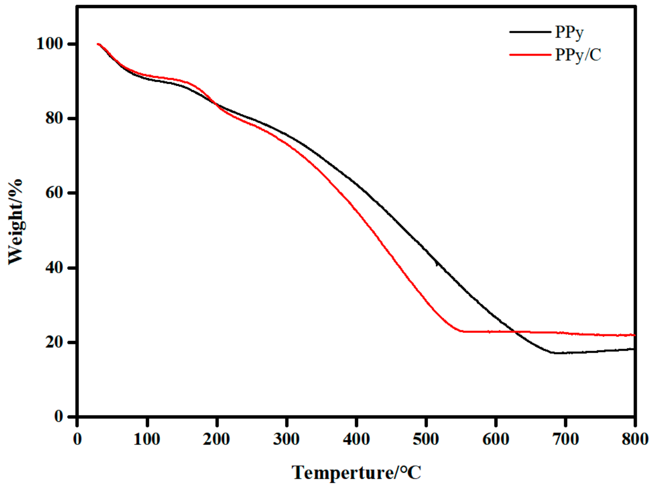

Fig. S3 TGA curve of (a) PPy, (b) PPy/C coatings under N_2 atmosphere.

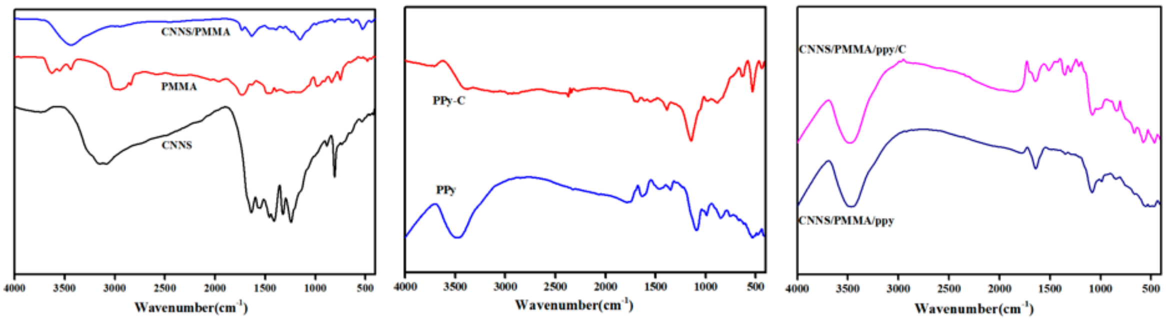

Fig. S4 FT-IR spectra of different coatings.

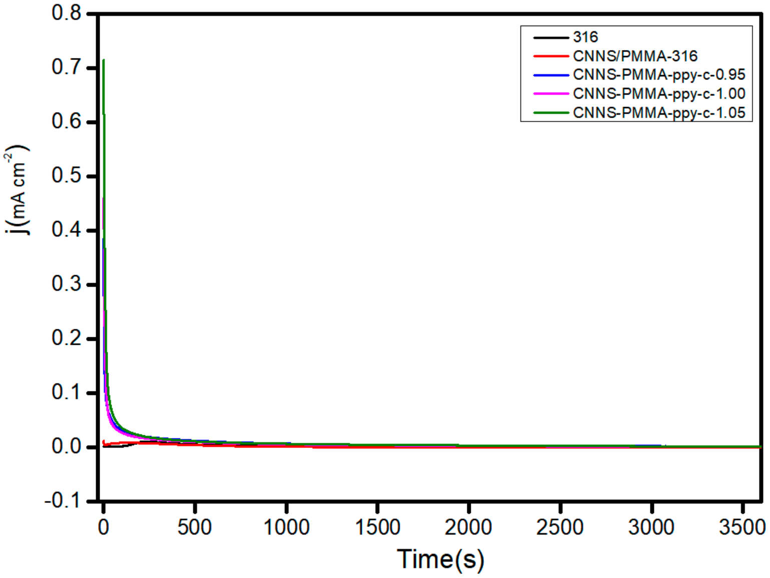

Fig. S5 Potentiostatic polarization curves for the bare 316SS and composite coatings in 0.1 M H_2SO_4+ 2 ppm HF at 70°C in 0.6V.

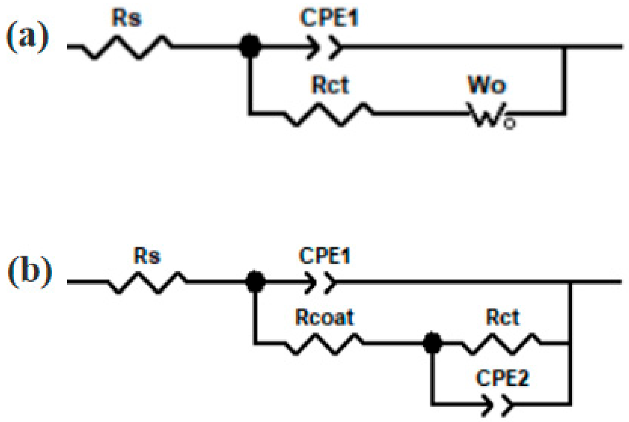

Fig. S6 Equivalent circuit for fitting the impedance diagram of (a)for bare 316SS and CNNS/PMMA-316, (b)for coated 316SS.

Table S1 The fitted electrochemical parameters of different samples

Samples	Rs ($\Omega\cdot cm^2$)	CPE Y_0 ($\Omega\cdot cm^{-2}\cdot S^{-n}$)	CPE n	R_{coat} ($\Omega\cdot cm^2$)
**316SS**	6.372	2.134×10^{-5}	0.749	5.532
**CNNS/PMMA-316**	5.051	1.364×10^{-3}	0.667	4.384

Samples	Rs ($\Omega\cdot cm^2$)	CPE1 Y_0 ($\Omega\cdot cm^{-2}\cdot S^{-n}$ $\times10^{-4}$)	CPE1 n	Rcoat ($\Omega\cdot cm^2$)	CPE2 Y_0 ($\Omega\cdot cm^{-2}\cdot S^{-n}$ $\times10^{-5}$)	CPE2 n	Rct ($\Omega\cdot cm^2$)
**PPy-0.80**	3.951	21.779	0.428	9.044	23.102	0.912	775.2
**PPy-0.90**	4.859	58.472	0.388	8.168	19.689	0.940	2068

**PPy-1.00**	4.576	16.145	0.638	5.937	17.777	0.928	1383
**PPy-1.10**	3.638	10.328	0.698	4.241	20.682	0.923	453.1
**PPy/C-0.80**	2.677	1.383	0.8822	3.606	18.129	0.922	2615
**PPy/C-0.90**	3.080	9.7728	0.689	3.987	22.693	0.928	907.4
**PPy/C-1.00**	4.372	17.951	0.673	6.824	19.518	0.921	2152
**PPy/C-1.10**	4.137	2.583	0.844	4.709	19.591	0.926	758.8
**CNNS/PMMA/ PPy/C-0.95**	5.955	18.069	0.5404	11.86	19.316	0.922	2468
**CNNS/PMMA/ PPy/C-1.00**	6.361	20.599	0.562	16.3	23.322	0.896	2802
**CNNS/PMMA/ PPy/C-1.05**	5.436	13.975	0.590	15.73	18.961	0.921	2188

Table S2 Interfacial contact resistance (ICR) values of different samples at a compaction force of 140 N/cm^2

**Sample**	**ICR ($m\Omega\cdot cm^2$)**
316	66
PPy	51
PPy/C	22
CNNS/PMMA/PPy	115
CNNS/PMMA/PPy/C	86

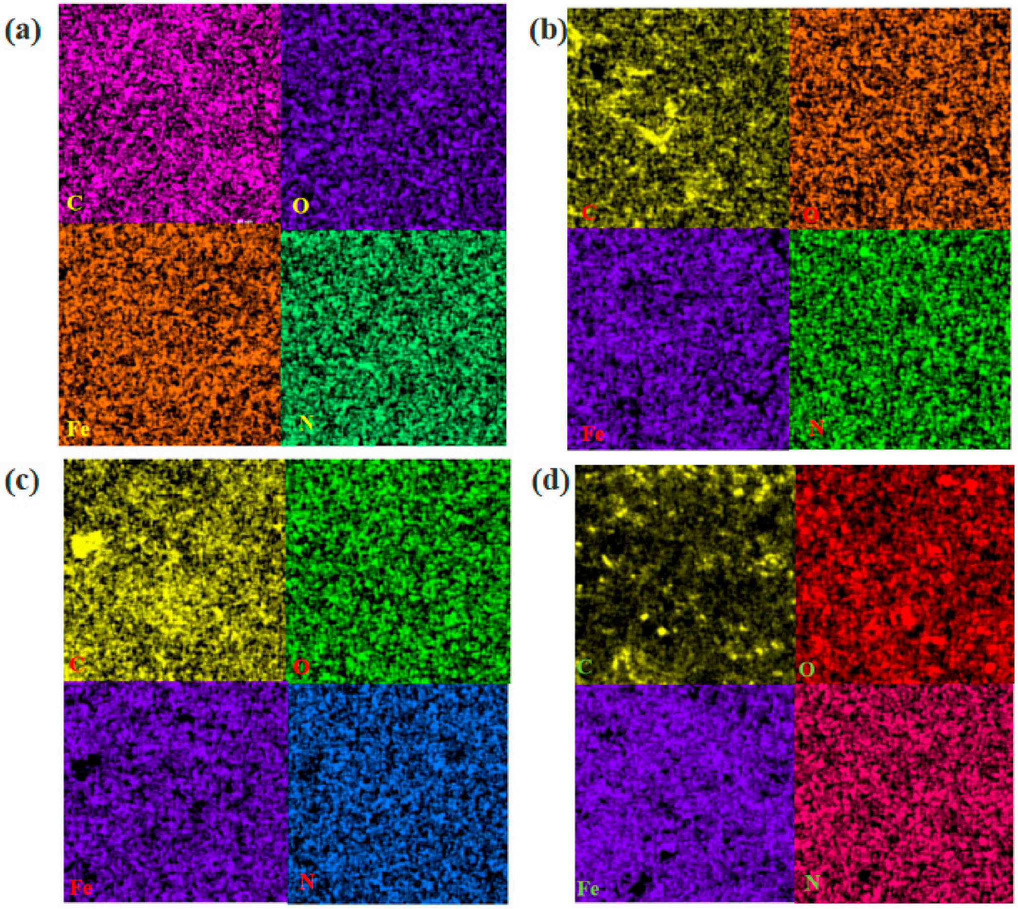

Fig. S7 EDX-mapping of (a)PPy, (b)PPy/C, (c)CNNS/PMMA/PPy, (d)CNNS/PMMA/PPy/C
